# Epigallocatechin-3-gallate enhances ER stress-induced cancer cell apoptosis by directly targeting PARP16 activity

**DOI:** 10.1038/cddiscovery.2017.34

**Published:** 2017-07-10

**Authors:** Juanjuan Wang, Chenggang Zhu, Dan Song, Ruiqi Xia, Wenbo Yu, Yongjun Dang, Yiyan Fei, Long Yu, Jiaxue Wu

**Affiliations:** 1 The State Key Laboratory of Genetic Engineering, Zhongshan Hospital and School of Life Science, Fudan University, Shanghai, PR China; 2 Department of Optical Science and Engineering, Shanghai Engineering Research Center of Ultra-Precision Optical Manufacturing, Key Laboratory of Micro and Nano Photonic Structures (Ministry of Education), Fudan University, Shanghai, PR China; 3 Key Laboratory of Metabolism and Molecular Medicine, The Ministry of Education, Department of Biochemistry and Molecular Biology, School of Basic Medical Sciences, Fudan University, Shanghai, PR China

## Abstract

Poly(ADP-ribose) polymerases (PARPs) are ADP-ribosylating enzymes and play important roles in a variety of cellular processes. Most small-molecule PARP inhibitors developed to date have been against PARP1, a poly-ADP-ribose transferase, and suffer from poor selectivity. PARP16, a mono-ADP-ribose transferase, has recently emerged as a potential therapeutic target, but its inhibitor development has trailed behind. Here we newly characterized epigallocatechin-3-gallate (EGCG) as a potential inhibitor of PARP16. We found that EGCG was associated with PARP16 and dramatically inhibited its activity *in vitro*. Moreover, EGCG suppressed the ER stress-induced phosphorylation of PERK and the transcription of unfolded protein response-related genes, leading to dramatically increase of cancer cells apoptosis under ER stress conditions, which was dependent on PARP16. These findings newly characterized EGCG as a potential inhibitor of PARP16, which can enhance the ER stress-induced cancer cell apoptosis, suggesting that a combination of EGCG and ER stress-induced agents might represent a novel approach for cancer therapy or chemoprevention.

## Introduction

ADP-ribosylation is a post-transcriptional modification catalyzed by a family of ADP-ribosyl transferases, the poly(ADP-ribose) polymerases (PARPs) in eukaryotic cells.^1^ The human PARP family comprises at least 18 members, which transfer ADP-ribose moiety from co-substrate *β*-NAD^+^ to acceptor proteins including themselves, leading to protein ADP-ribosylation and regulating the functions of the modified proteins.^2^ Among this family, PARP1 is best-characterized and involved in a variety of cellular functions.^3^ PARP1 inhibitors have been extensively investigated for the treatment of various cancer types.^4,5^ Inhibition of the catalytic activity of PARP1 was found to result in synthetic lethality in BRCA1/2-deficient cancer cells.^6,7,8^ This cancer therapy strategy was clinically established by the approval of Olaparib for treatment of the advanced ovarian cancer patient containing germline BRCA mutation.^9,10^

Besides poly-ADP-ribosyl transferases, a large subset of the PARP family consists of mono-ADP-ribosyl transferases that are thought to modify themselves and targets by covalently adding only a single ADP-ribose moiety.^1^ Emerging evidences suggest that some of these mono-ADP-ribosyl transferases play important roles in cellular functions. For instance, PARP14 has been found to play important roles in DNA damage response, T-cell development, macrophage activation and tumor development.^11,12,13,14^ PARP10 was demonstrated as a component in the NF-*κ*B signaling pathway by directly modifying NEMO.^15^ Most of the mono-ADP-ribosyl transferases remain poorly characterized, in large part due to a lack of small-molecule inhibitors.

To date, the majority of current PARP inhibitors are nicotinamide mimics that display broad inhibition of poly-ADP-ribosyl transferases,^16^ and inhibit the mono-ADP-ribosyl transferases with low potency.^17^ Recently, inhibitors against some of the mono-ADP-ribosyl transferases, including PARP10 and PARP14, were identified and characterized by high-throughput screening or other strategy.^17,18,19, 20^

PARP16 is the only known PARP with a putative C-terminal trans-membrane domain associated with the nuclear envelope and the endoplasmic reticulum (ER).^21,22^ PARP16 has been found to be required for activating the functionally related ER stress sensors PERK and IRE1*α*, suggesting that PARP16 plays a critical role in regulating the unfolded protein response (UPR) of the ER.^23^ More recently, Angelica *et al.*^24^ demonstrated that PARP16 was required for the formation of stress assemblies in Drosophila, and linked mono-ADP-ribosylation to a metabolic stress. These results indicate that PARP16 plays a critical role in response to stress.

In recognition of the urgent need to develop small-molecule inhibitors against PARP16 and other less-studied PARPs, we reported herein a small-molecule microarray-based strategy for high-throughput screening of potential inhibitors of PARP16. Finally, we demonstrated that epigallocatechin-3-gallate (EGCG) was a potential inhibitor of PARP16, which suppressed the ER stress-induced phosphorylation of PERK and the transcription of UPR-related genes, leading to a dramatical increase of the cancer cells apoptosis under ER stress conditions.

## Results

### Identification of potential inhibitors against PRAP16

**Figure 1:**
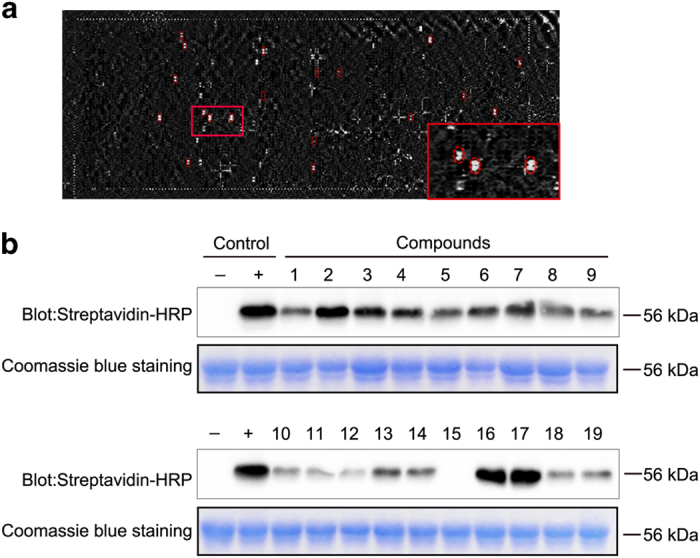
Identification of potential inhibitors of PARP16. (**a**) The difference OI-RD image of small-molecule microarrays including 3375 compounds before and after reaction with PARP16 protein. (**b**) Relative inhibitory activity of 19 compounds against PARP16 *in vitro.*

**Figure 2:**
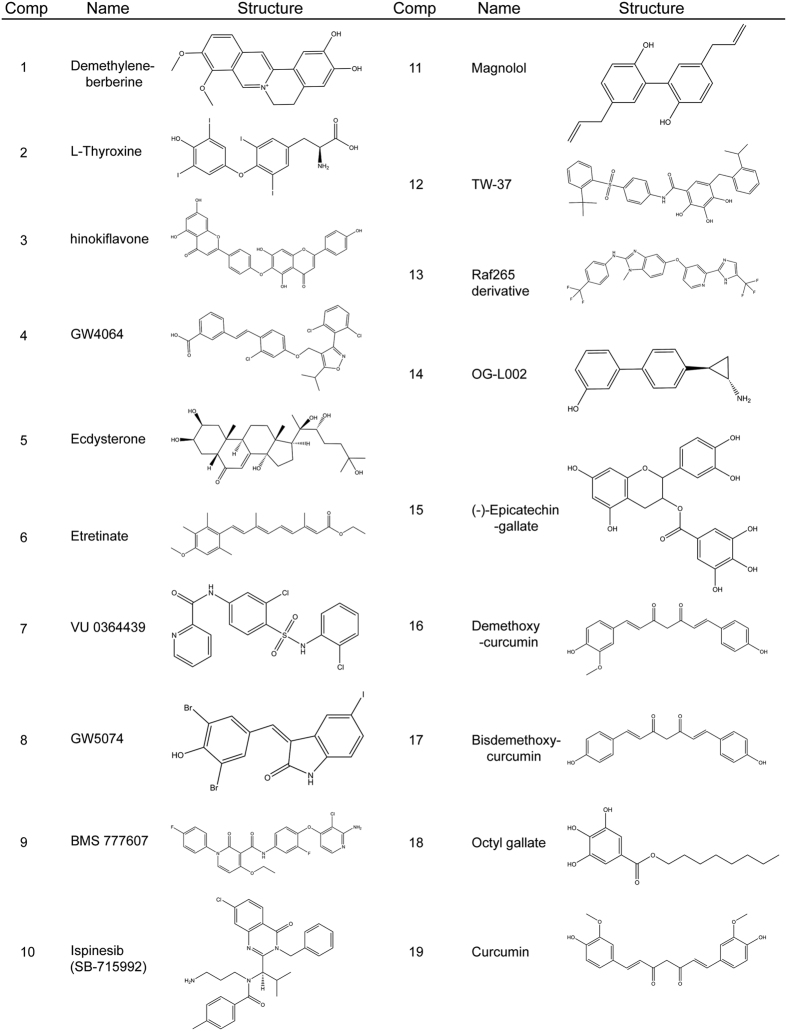
Names and structures of compounds 1–19.

To screen the potential inhibitors against PARP16, the GST-PARP16 was expressed in *Escherichia coli* and purified using affinity chromatograph. As shown in Supplementary Figure S1A, a single band with a molecular weight of 56 kDa was observed by Coomassie brilliant blue staining. The enzyme activity of purified GST-PARP16 was examined by *in vitro* ADP-ribosylation assay using biotinylated NAD^+^ as the substrate, followed by western blotting using Streptavidin-HRP. Consistent with previous reports, GST-PARP16 can modify itself *in vitro* as detected a strong single band by Streptavidin-HRP (Supplementary Figure S1B).^23^ The GST-PARP16 protein was used to identify its binding partners based on small-molecule microarray and OI-RD optical biosensor screening as described in Materials and Methods. Microarray containing 3375 compounds was screened and 19 small molecules were found to have high affinity association with PARP16 (Figure 1a and Figure 2). Then the activity of PARP16 was examined *in vitro* at the presence of indicated small molecules. As shown in Figure 1b, most of the selected 19 small molecules inhibited the auto-ADP-ribosylation of PARP16 *in vitro* at the concentration of 0.5 mM for each compound. Among them, the compound 15 totally abolished the activity of PARP16, suggesting that it may be a potential inhibitor of PRAP16.

### EGCG and epicatechin-3-gallate inhibited the PARP16 activity

**Figure 3:**
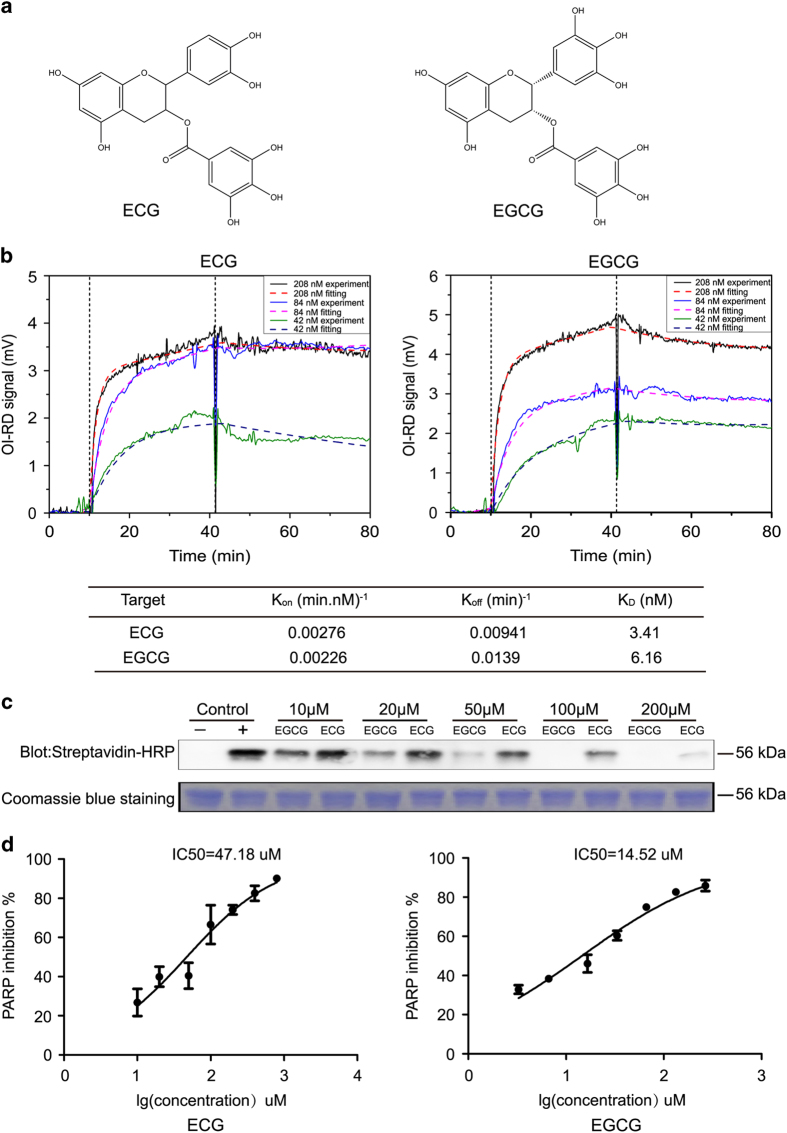
EGCG inhibited PARP16 activity *in vitro*. (**a**) Chemical structures of ECG and its analog EGCG. (**b**) Binding curves of surface immobilized ECG and EGCG with flowing PARP16 at respective concentrations of 208, 84 and 42 nM. Vertical lines marked the starts of association and dissociation phases of the binding event. The dash lines were global fits to a Langmuir reaction model with the global fitting parameters listed at the bottom of the curves. (**c**) Concentration-dependent activity tests of ECG and EGCG against PARP16, which were detected by western blot with Streptavidin-HRP after reactions. The concentrations of ECG and EGCG in the experiments were from 10 to 200 *μ*M. (**d**) The IC_50_ values were determined from dose–response curves using eight concentrations of each compound in triplicate based on ELISA assay. Curves were fitted to data points using nonlinear regression analysis and IC_50_ values were interpolated from the resulting curves using Graphpad prism 5 software. Data were shown as means±S.D. for the independent experiments.

Compound 15 is epicatechin-3-gallate (ECG), the third major catechin component in green tea, which has been shown strong biological activity in some aspects, including apoptosis, cell growth inhibition in various cells.^25^ However, the most abundant and powerful antioxidant in green tea for cancer chemoprevention is EGCG.^26^ Interestingly, ECG and EGCG have very similar structures (Figure 3a), raising the possibility that EGCG may also inhibit the PARP16 activity. To test the possibility, we firstly compared the binding affinities of ECG and EGCG with PARP16. We measured binding kinetics of PARP16 to immobilized ECG and EGCG during association and dissociation phases and extracted reaction rate constants from these curves as described in Materials and Methods. By repeating the binding reaction of PARP16 at concentrations of 208, 84 and 42 nM on separate fresh microarrays, binding curves of ECG and EGCG at different probe concentrations were recorded (Figure 3b). Then, the binding curves were fitted to yield the reaction kinetic rate constants using the Langmuir reaction model. The binding kinetic constants revealed that PARP16 bound ECG or EGCG with a dissociation constant (Kd) of 3.41 and 6.16 nM, respectively, suggesting that it had high binding affinities with both ECG and EGCG (Figure 3b).

Then we examined the inhibition efficiency of ECG and EGCG against PARP16 activity by *in vitro* ADP-ribosylation assay. To our surprise, although the binding affinity of EGCG was weaker than ECG, EGCG can inhibit the PARP16 activity more effectively than ECG under the same conditions *in vitro* (Figure 3c). Moreover, the IC_50_ of EGCG and ECG against PARP16 activity were 14.52 and 47.18 *μ*M, respectively (Figure 3d). Taken together, these results indicated that EGCG was a potential inhibitor of PARP16.

### EGCG suppressed the phosphorylation of PERK induced by ER stress

PARP16 has been shown to be required for activating the functionally related ER stress sensors PERK and IRE1*α* during the UPR.^23^ To further confirm that, PARP16-deficient QGY-7703 cells were generated by CRISPR-Cas9 system as described. A sgRNA was designed to target exon 1 of PARP16 (Supplementary Figure S2A). PARP16-deficient QGY-7703 cell lines were established, which totally lost the PARP16 protein as examined by western blotting using anti-PRAP16 antibody (Supplementary Figure S2C). The targeting regions of PARP16-deficient cells were amplified by PCR and Sanger sequencing of PCR products demonstrated that PARP16-deficient cells contained deletion of several base pairs in exon 1 of PARP16, respectively (Supplementary Figure S2B). Then the phosphorylation level of PERK and its downstream substrate eIF2*α* induced by ER stress were examined in PARP16 wild type and deficient cells. As shown in Supplementary Figure S2D, the phosphorylation of PERK and eIF2*α* were dramatically induced by Brefeldin A (BFA) treatment in wild-type cells, but not in PARP16-deficient cells. These results were consistent with previous report,^23^ and indicated that PARP16 was essential for the PERK activation under ER stress condition.

**Figure 4:**
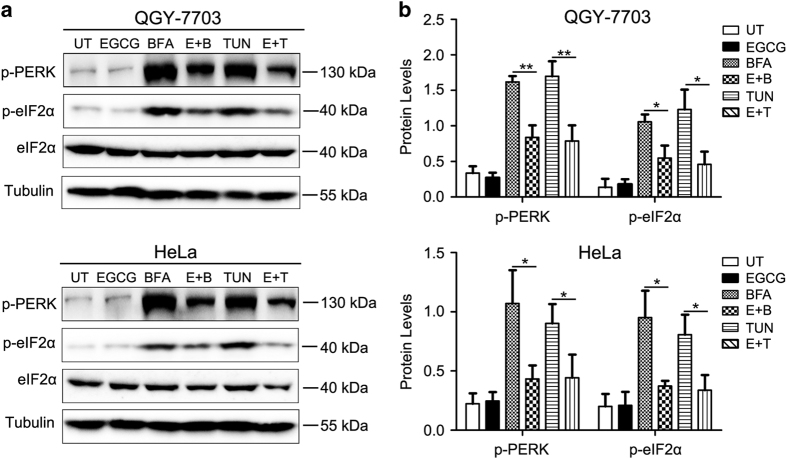
EGCG suppressed the ER stress-induced PERK signaling. (**a**) QGY-7703 or Hela cells were pre-treated with or without 100 *μ*M EGCG for 2 h followed by UPR induction for 6 h. Cell lysates were resolved by SDS-PAGE and then immunoblotted with antibodies against p-PERK (Thr 981), p-eIF2*α* (Ser 51), eIF2*α* (total) and Tubulin. (**b**) Relative protein levels of p-PERK and p-eIF2*α* were normalized against tubulin by Image J Analysis Software and data were shown as means±S.D. for three independent experiments. *P<0.05, **P<0.01. BFA, Brefeldin A treated; p-PERK, phospho-PERK; p-eIF2*α*, phospho-eIF2*α*; TUN, Tunicamycin treated**; UT, untreated.

The activity of PARP16 was inhibited by EGCG, raising the possibility that EGCG may also suppress the phosphorylation of PERK induced by ER stress. To test this hypothesis, the phosphorylation of PERK and eIF2*α* were examined by treatment of QGY-7703 and Hela cells with EGCG, Tunicamycin (TUN) and BFA alone or EGCG in combination with BFA or TUN. As shown in Figure 4a and b, compared with control cells, the phosphorylation of PERK and eIF2*α* were dramatically induced by BFA and TUN, and this induction was effectively suppressed by EGCG treatment. These results indicated that EGCG suppressed the phosphorylation of PERK and eIF2*α* induced by ER stress.

### EGCG attenuated the transcriptions of UPR-related gene induced by ER stress

**Figure 5:**
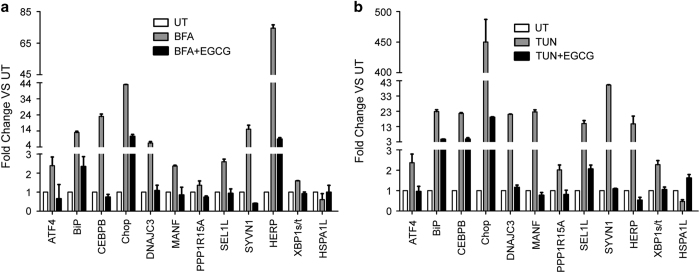
EGCG attenuated the expression of ER stress-induced UPR-related genes. RT-qPCR analysis of UPR-dependent transcription in Hela cells pre-treated with or without 100 *μ*M EGCG for 2 h followed by BFA (**a**) or TUN (**b**) treatment for another 6 h. The normalized values were then calibrated against the control value, data represented mean fold change in RNA expression of genes and were shown as means±S.D. for the independent experiments.

Activation of PERK and IRE1*α* signaling activated downstream transcription factors leading to change of the expression of UPR-related genes. Then the expression of UPR-related gene was examined by quantitative real-time PCR after treatment with EGCG, TUN and BFA alone or EGCG combined with BFA or TUN in Hela cells. As shown in Figures 5a and b, compared with control cells, the expression of UPR-related gene was dramatically induced by BFA and TUN, and this induction was suppressed by treatment of Hela cells with EGCG, further suggesting that EGCG suppressed the UPR induced by ER stress.

### EGCG enhanced the cell apoptosis induced by ER stress

**Figure 6:**
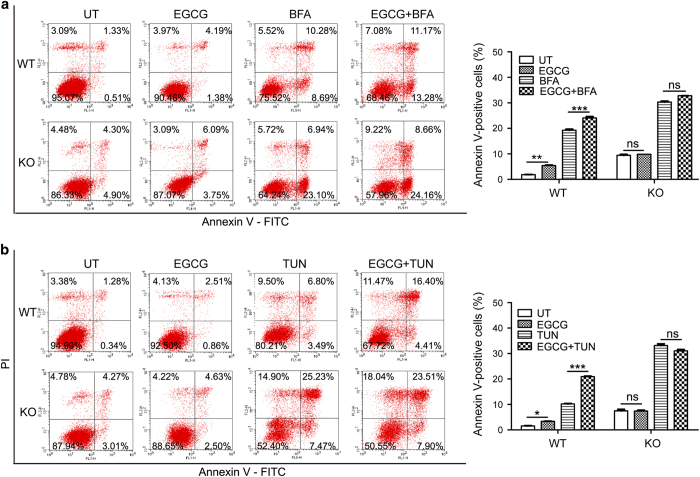
EGCG enhanced ER stress-induced apoptosis by targeting PARP16. Flow cytometry analysis with Annexin V-PI staining was performed to evaluate the percentage of apoptotic cells in EGCG combination with BFA (**a**) or TUN (**b**) or BFA/TUN treatment alone induced QGY-7703 WT and PARP16-deficient cells for 24 h. EGCG treatment significantly increased the percentage of apoptotic cells in the QGY-7703 WT cells when compared with that of BFA or TUN treatment alone. While EGCG played little or no role in the percentage of apoptotic cells in the PARP16-deficient cells compared with that of controls. Histograms showing analysis on cell apoptosis results were displayed on the right and data were shown as means±S.D. for the independent experiments. *P<0.05, **P<0.01, ***P<0.001 and NS indicated there was not statistically significant (P>0.05). BFA: 5 *μ*g/ml; TUN: 5 *μ*g/ml; EGCG: 100 *μ*M.

It has been found that PARP16 knockdown rendered cells highly sensitive to ER stress, resulting in an increased level of cell death. To further confirm that, we examined the apoptosis of PARP16 wild type or deficient cells after treatment with BFA by flow cytometry using FITC-Annexin V/PI apoptosis detection kit. As shown in Supplementary Figure S3, compared with wild-type cells, the Annexin V-positive cells were dramatically increased in PARP16-deficient cells after BFA treatment, which was consistent with previous report.^23^ Then we examined the effect of EGCG on TUN or BFA induced cell apoptosis in PARP16 wild type and deficient cells. As shown in Figure 6, EGCG treatment only induced a less than 10% apoptosis in PARP16 wild-type cells. However, EGCG treatment only could not further augment the apoptosis of PARP16-deficient cells. Moreover, EGCG treatment dramatically increased the Annexin V-positive cells induced by TUN or BFA in wild-type cells. On the contrary, EGCG treatment could not increase the Annexin V-positive cells induced by TUN or BFA in PARP16-deficient cells (Figure 6). These results indicated that EGCG enhanced the ER stress-induced cell apoptosis by targeting PARP16 activity.

## Discussion

Green tea is one of the most ancient and widely consumed beverages in the world. Previous studies have shown that consumption of green tea has benefits for treating human diseases, such as Parkinson’s disease and cancer.^27,28,29^ The major flavonoids of green tea extracts are catechins, including epicatechin (EC), epigallocatechin (EGC), epicatechin-3-gallate (ECG) and EGCG.^30^ Among them, EGCG is the most abundant component and has been shown to possess a wide range of pharmacological properties, including chemopreventive, anticarcinogenic, anti-infective and antioxidant activity.^31,32,33^ The antineoplastic activity of EGCG has also been widely investigated in cell culture, animal models and clinical studies.^34^ Previously we have shown that EGCG can inhibit carbonyl reductase 1 (CBR1) activity and enhance the effectiveness and decrease the cardiotoxicity of the anticancer drug daunorubicin (DNR), suggesting that a combination of EGCG and DNR might represent a novel approach for hepatocellular carcinoma therapy or chemoprevention.^35^ In this study, we found that ECG was a binding partner of PARP16 by high-throughput screening using a small-molecule microarray-based strategy. The enzymatic activity of PARP16 was dramatically inhibited by ECG *in vitro* (Figure 1). The structures of ECG and EGCG are very similar, both containing a gallate moiety compared with EC and EGC. Although both ECG and EGCG can potentially inhibit the proliferation and induce apoptosis of cancer cells, EGCG is reportedly the most promising and is under clinical investigation in chemoprevention trials.^34^ Our results also indicated that EGCG inhibited the PARP16 activity more effectively than ECG, although the binding affinity between EGCG and PARP16 was weaker than the affinity between ECG and PARP16 (Figure 3).

Accumulating evidences have implicated that UPR, an ER stress sensing/repair pathway, is involved in cell survival and tumor progression.^36,37^ The purpose of UPR is to balance the ER folding environment under ER stress.^38^ If ER stress is prolonged and the UPR fails to restore ER homeostasis, tumor cells will undergo cell death.^39^ The importance of UPR in the maintenance of malignancy has inspired great interest in exploring the therapeutic potential of targeting UPR components.^40,41,42^ For example, Irestatin and GSK2656157 have been found to inhibit the activity of IRE1 and PERK, the primary effectors of the UPR, to enhance the ER stress-induced apoptosis of cancer cells.^43,44,45^ In this study, we have demonstrated that EGCG can dramatically inhibit the activity of PARP16, and then suppressed the ER stress-induced PERK phosphorylation, leading to dramatical increase of the ER stress-induced apoptosis of cancer cells. These results indicate that EGCG can be used in combination with ER stress-induced drugs to treat the cancer cell.

The ability to induce cancer cell apoptosis of EGCG has been demonstrated in different cancer cell lines.^46,47,48^ However, the underlying mechanism is poorly understood. In this study, we have found that EGCG induced the apoptosis in PARP16 wild-type cells treated with or without ER stress inducers, but not in PAPP16-deficient cells, suggesting that EGCG-induced apoptosis was mediated by PARP16 both in normal conditions and ER stress conditions. Interestingly, EGCG had previously been found to bind to the ATP-binding domain of glucose regulate protein 78 (GRP78), blocking its UPR protective function and sensitizing glioma cells against chemotherapeutic agents such as etoposide.^49^ These findings indicated that EGCG may suppress the UPR signaling through different ways.

The role of ER stress response in cancer was initially proposed in 2004 by Ma and Hendershot, which suggested that ER stress signaling play an important role in tumor progression and survival either by eliminating the stressful trigger (such as hypoxia, nutritional stress) or by adapting to it. However, if these countermeasures prove unsuccessful and severe imbalances persist, ER stress response abandon its survival function and instead initiate proapoptotic mechanisms that induce cell death.^50,51,52^ Therefore, this differential may represent an opportunity for cancer therapy aimed at the already engaged ER stress and inhibition of the ER stress signaling. In particular, tumor-specific blockage of the PERK signaling by EGCG, and strong stimulation of ER stress by TUN or BFA, might serve to provide meaningful therapeutic benefit.

In conclusion, we identified that PARP16 was a new target of EGCG. EGCG suppressed the activity of PARP16, then blocked the ER stress-induced UPR signaling and increased the apoptosis of cancer cells. These findings also indicated that a combination of EGCG and ER stress-induced drug might represent a novel approach for cancer therapy or chemoprevention.

## Materials and methods

### Cell lines

Hela and QGY-7703 cells were obtained from Shanghai Cell Bank of Chinese Academy of Sciences (Shanghai, China). Cells were cultured in DMEM medium (Hyclone, Logan, UT, USA) supplemented with 10% (v/v) fetal bovine serum (Gibco, Carlsbad, CA, USA), 100 U/ml penicillin and 100 μg/ml streptomycin (Euroclone, SpA, Milan, Italy), at 37 °C in a 5% CO_2_ atmosphere.

### Plasmids, antibodies and other materials

PARP16 was cloned into pGEX-4T-1 vector and confirmed by sequencing. Anti-phos-PERK (Thr 981) and anti-*β*-Tubulin antibodies were purchased from Santa-Cruz Biotechnologies (Dallas, TX, USA); anti-eIF2*α* and anti-phos-eIF2*α* (Ser 51) antibodies were purchased from Abcam (Cambridge, MA, USA), anti-PARP16 antibody was generated by ourselves; Streptavidin-HRP was obtained from Thermo Fisher (Waltham, MA, USA); BFA, ECG and EGCG were purchased from Sigma-Aldrich (St Louis, MO, USA); TUN was obtained from Cell Signaling Technologies (Beverly, MA, USA); Biotin-labeled NAD^+^ from Invitrogen (Carlsbad, CA, USA); glutathione Sepharose 4B resin was obtained from GE Healthcare (Pittsburgh, PA, USA).

### Fabrication of small-molecule microarrays

The small-molecule library consists of 3375 bioactive compounds, including 1053 natural compounds from Traditional Chinese Medicine (most of them from herbs), 1527 drugs approved by Food and Drug Administration and 795 known inhibitors. In total, 3375 bioactive compounds were printed on phenyl-isocyanate-functionalized glass slides and each compound was printed in duplicate. After printing, small-molecule microarrays were dried at 45 °C for 24 h to facilitate covalent bonding of nucleophilic groups of small molecules to isocyanate groups of the slides, as described before.^53^

### Preliminary screening of lead compounds for PARP16

Preliminary screening of lead compounds was performed by incubating small-molecule microarrays with PARP16 and detecting binding results with a microarray compatible label-free detection instrument, oblique-incidence reflectivity difference (OI-RD) microscope, which is able to study more than 10 000 biomolecular interactions in a single experiment without the need to label any biomolecules.^54,55,56^ For the preliminary screening, as-prepared small-molecule microarray was assembled into a fluidic cartridge and washed *in situ* with a flow of 1×PBS buffer (WISENT) to remove excess unbound small molecules. The small-molecule microarray was then blocked with 7600 nM BSA in 1×PBS for 30 min, followed by incubation with PARP16 at a concentration of 208 nM for 2 h. The compounds which reacted with PARP16 were determined as bright doublets in the differential OI-RD image.

### ADP-ribosylation assay *in vitro*

GST-PARP16 was incubated with PARylation buffer (100 mM Tris-HCl (pH7.6), 12.5 *μ*M biotinylated NAD^+^, 10 mM MgCl_2_, 50 *μ*g DNA octamer (5′- GGAATTCC-3′) and 10 mM DTT) for 30 min at 30 °C, with or without the compounds. Then, the samples were analyzed by immunoblotting with Streptavidin-HRP.

### Binding kinetics measurement of PARP16 to ECG and EGCG

With an OI-RD microscope, we measured binding kinetics of PARP16 to immobilized ECG and EGCG during association and dissociation phases and extracted reaction rate constants from these curves as follows. ECG and EGCG were printed in triplicate on isocyanate-functionalized glass slides at respective concentrations of 11.3 and 10.9 mM, and six identical microarrays were fabricated on each glass slide. The slide was assembled into a fluidic cartridge with six independent chambers with each of microarray housing in a separate chamber. Before binding reaction, the slide was washed *in situ* with a flow of 1×PBS to remove excess unbound small molecules, followed by blocking with 7600 nM BSA in 1×PBS for 30 min. For binding kinetics measurement, 1×PBS was first flowed through a reaction chamber at a flow rate of 0.01 ml/min for 10 min to acquire the baseline. Next, 1×PBS was quickly replaced with PARP16 solution at a flow rate of 2 ml/min followed by a reduced flow rate at 0.01 ml/min to allow the microarray to be incubated in the PARP16 solution under the flow condition for 30 min (association phase of the reaction). Finally, the PARP16 solution was quickly replaced with 1×PBS at 2 ml/min followed by a flow rate of 0.01 ml/min to allow protein PARP16 to dissociate for 40 min (dissociation phase of the reaction). By repeating the binding reaction of protein PARP16 at concentrations of 208, 84 and 42 nM on separate fresh microarrays, binding curves of ECG and EGCG at different probe concentrations were recorded. Afterward, the binding curves were fitted to yield the reaction kinetic rate constants using the Langmuir reaction model as described in Landry *et al.*^56^

### Measurement of IC_50_ by enzyme-linked immunosorbent assay

The IC_50_ of ECG and EGCG to PARP16 were measured by enzyme-linked immunosorbent assay (ELISA). Micro-ELISA plate was coated with 100 *μ*l (10 *μ*g/ml) PARP16 protein as a substrate overnight at 4 °C. Then discard whole liquid and dry followed by adding 12.5 *μ*M biotin-labeled NAD^+^, PARylation buffer and various concentrations of ECG or EGCG. Meanwhile, there were two controls, one without any protein or inhibitor and another with protein and no inhibitor. The control values were included as two log points above and below the graph assigned for conditions where there was 0 and 100% activity. For each concentration, compounds were tested with five replicates on the plate and three separate experiments were carried out for each compound. The reactions lasted at 30 °C for 1 h. Upon completion, each well was washed twice and then incubated with Streptavidin-HRP, TMB Substrate Reagent and Stop Solution sequentially. Optical density was detected by a 96-well multiscanner autoreader at 450 and 630 nm wave lengths. IC_50_ values were determined by nonlinear regression using the GraphPad Prism 5 software (GraphPad Prism, La Jolla, CA, USA).

### Generation of PARP16-deficient cells

PARP16-deficient cells were generated by using the CRISPR-Cas9 system. Briefly, QGY-7703 cells were transiently transfected with sgRNA targeting PARP16 and expressed from the pX335-U6-Chimeric-BB-CBh-hSpCas9n vector containing Cas9 followed by the 2A-Puromycin cassette. The next day, cells were selected with puromycin for 2 days and subcloned to form single colonies. Clones were screened by immunoblot to verify the loss of PARP16 expression and subsequently characterized by PCR and sequencing. The genomic region targeted by the CRISPR-Cas9 was amplified and the PCR product was cloned into the T-vector before sequencing.

### Western blot

For analysis of the phosphorylation levels of PERK and eIF2*α*, cells were seeded in 12-well plates. After 24 h from seeding, cells were pre-incubated or not with EGCG at 100 *μ*M concentration for 2 h and then treated with 5 *μ*g/ml BFA or TUN for an additional 6 h. Then cells were harvested and re-suspended in NETN buffer (20 mM Tris-HCl (pH 8.0), 100 mM NaCl, 0.005 g/ml NP-40, 1 mM EDTA) supplemented with protease inhibitor cocktail for 15 min on ice. After centrifugation, we collected the supernatant and then denatured at 98 °C for 10 min. Protein samples were separated by SDS-PAGE and then transferred onto polyvinylidene difluoride filter membranes. After blocking, the membranes were incubated with specific antibodies against different proteins at 4 °C overnight followed by incubation with secondary antibodies, and finally detected via the infrared imaging system.

### RNA isolation, cDNA synthesis and quantitative real-time PCR (qRT-PCR)

Cellular total RNA was extracted using Trizol reagent (Invitrogen), and applied for reverse transcription using an oligo dT primer (Invitrogen) and reverse transcriptase (Invitrogen). qRT-PCR analysis was conducted using SYBR Green Supermix kit (Toyobo, Osaka, Japan) with a Light Cycler480 II (Roche, Basel, Switzerland). Properly diluted cDNA was used in a 10-μl qRT-PCR in triplicate for each gene. The cycle parameters were 95 °C for 1 min and 44 cycles of 95 °C for 10 s, 60 °C for 10 s and 72 °C for 20 s. Blank controls with no cDNA templates were performed to rule out contamination. A melting curve was obtained at the end of the PCR reaction to verify that only one product was produced. The relative gene expression levels normalized by *β*2-microglobulin (*β*2MG) were calculated by the formula 2^−ΔCt^, where the ΔCt (critical theshold)=Ct of genes of interest−Ct of *β*2MG. Fold changes of gene expression levels in the treatment groups relative to the untreated group were calculated by the 2^−ΔΔCt^ method, where ΔΔCt=ΔCt-treatment−ΔCt-untreatment. Statistical analysis was performed using the GraphPad Prism software. A two-tailed Student’s *t*-test was used to evaluate the group-level differences. All primers used in this study are given in Supplementary Table S1.

### The PI and annexin V staining for cell apoptosis detection

QGY-7703 cells were harvested after treatment with BFA (5 *μ*g/ml) or TUN (5 *μ*g/ml) or EGCG (100 *μ*M) alone or EGCG in combination with BFA or TUN and stained with the Annexin V/PI Apoptosis Detection Kit (BD) according to the manufacturer’s instructions. Data acquisition and analysis were performed with a FACS Calibur flow cytometer using CellQuest software (BD Biosciences, Franklin Lakes, NJ, USA).

### Statistics

All data were expressed as the mean±standard deviation (S.D.). The data shown in the study were obtained in at least three independent experiments performed in a parallel manner. Statistical analysis was performed using a two-tailed Student’s *t*-test. Probability values of less than 0.05, 0.01 and 0.001 were considered as statistically significant and marked with '*', '**' and '***' in respective figures.
