# Supplementary material for: Epigallocatechin-3-gallate enhances ER stress-induced cancer cell apoptosis by directly targeting PARP16 activity
Source: Cell Death Discov. 2017 Jul 10;3:17034–. doi: 10.1038/cddiscovery.2017.34 (PMC5502302; doi:10.1038/cddiscovery.2017.34)
Supplement: Supplementary Information [file cddiscovery201734-s1.docx]

**Supplementary Information**

**Supplementary Figure legends**

**Figure S1. PARP16 was auto-ADP-ribosylated in vitro.** (A) The purification of recombinant GST-PARP16 protein. Coomassie brilliant blue staining was shown. (B) PARP16 was auto-ADP-ribosylated in vitro by using biotinylated-NAD^+^ as the substrate.

**Figure S2. PARP16-deficient suppressed the ER stress-induced phosphorylation of PERK and eIF2α.** (A) sgRNA was designed to target the exon 1 of PARP16. (B) The genomic region of PARP16 in PARP16-deficient QGY-7703 cells were amplified by genomic PCR and analyzed by Sanger sequencing. The sequence results of QGY-7703 cells were shown. (C) The PARP16-deficient clones were identified by western blot using PARP16 antibody, where tubulin was used as loading control. (D) The phosphorylation of PERK and eIF2α were examined by western blotting in PARP16 wild type and deficient cells after treatment with 5 µg/ml BFA for 6 h.

**Figure S3. PARP16-deficient increased the ER stress-induced apoptosis of cancer cells.** (A) PARP16 wild type and deficient cells are treated with BFA for indicated times. The apoptotic cells were flow cytometric using Annexin V kit. (B) A histogram representing the percentage of Annexin V positive cells. Data were shown as means ± SD.

**
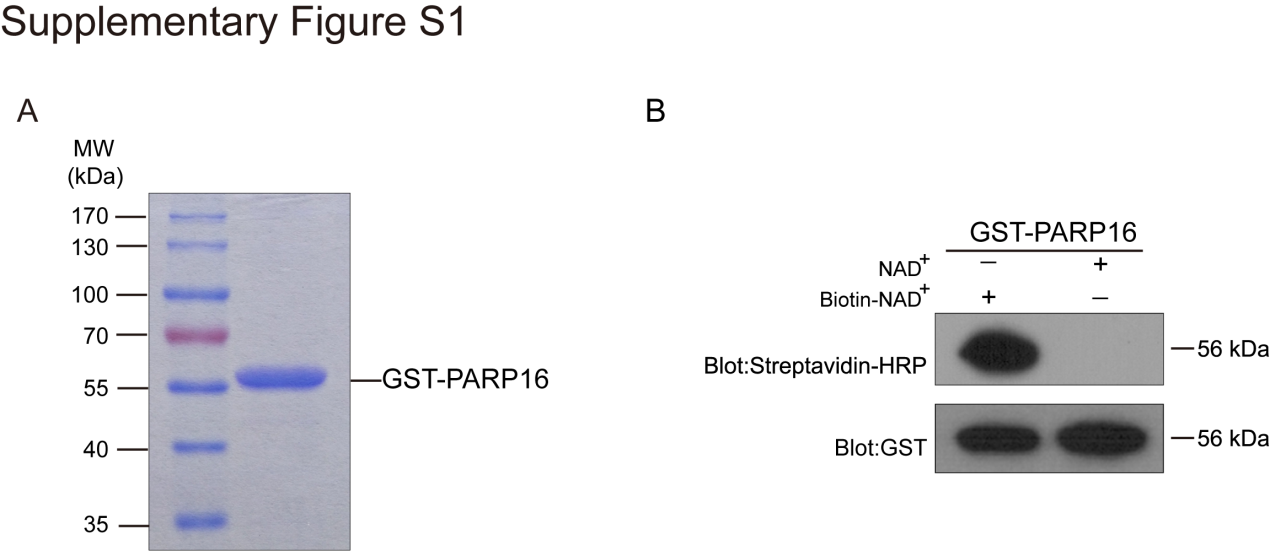
**

**
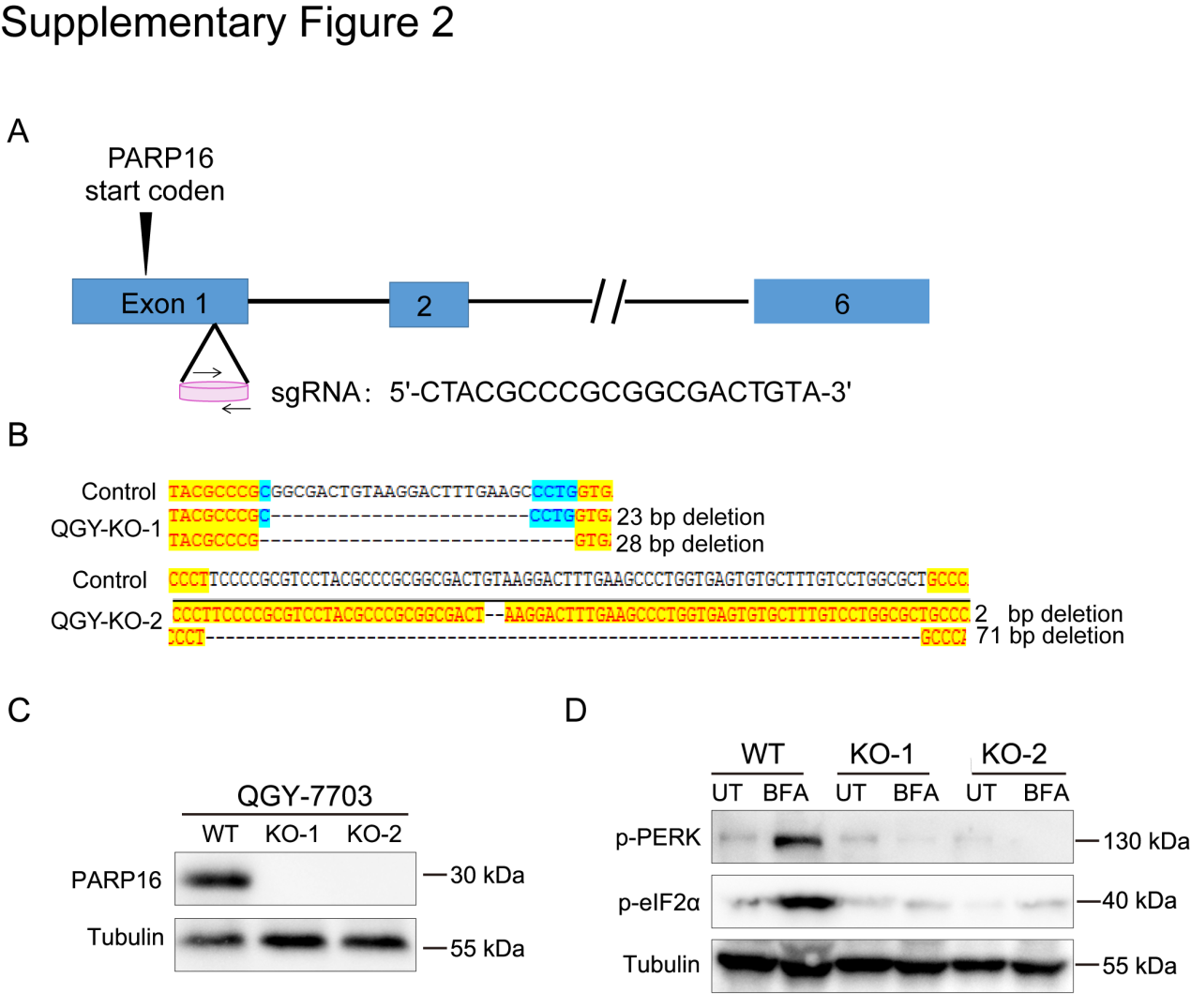
**

**
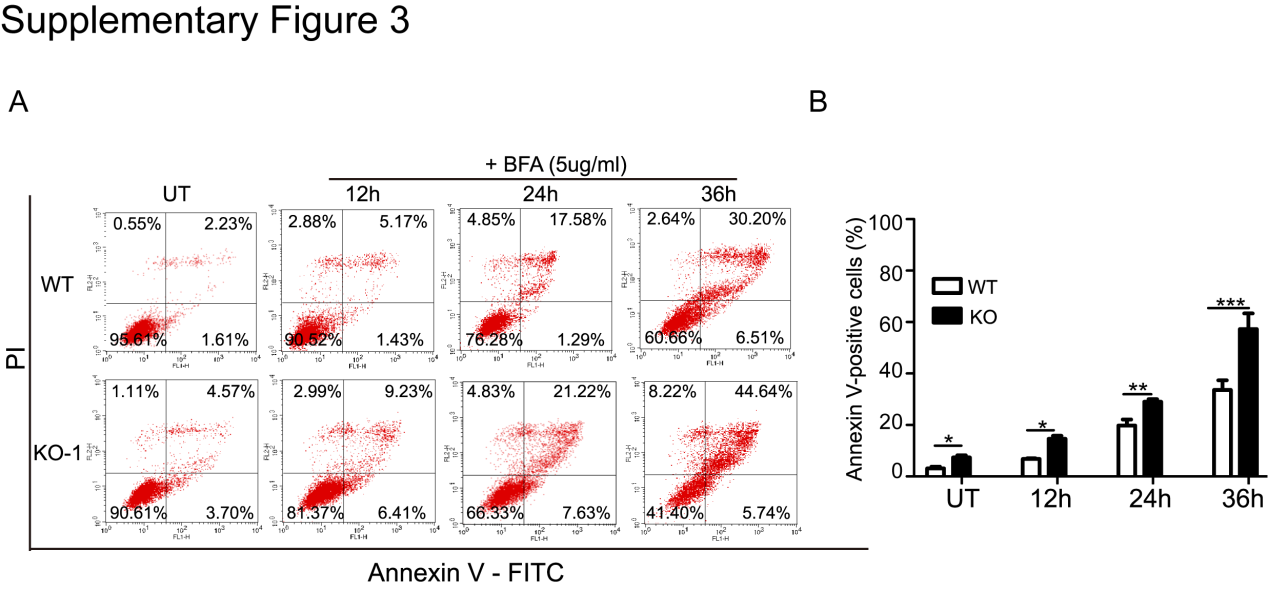
**

**Original Images**

**Figure 1**

**
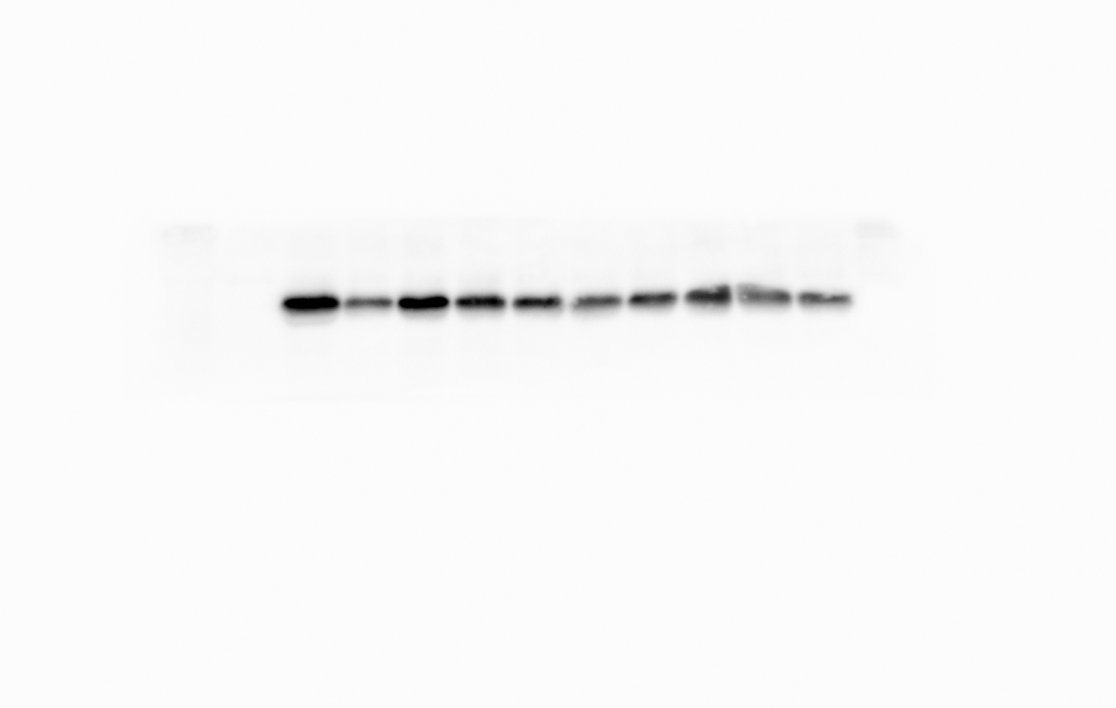
**

WB, Steptavidin-HRP-Comp1-9, Fig1B

**
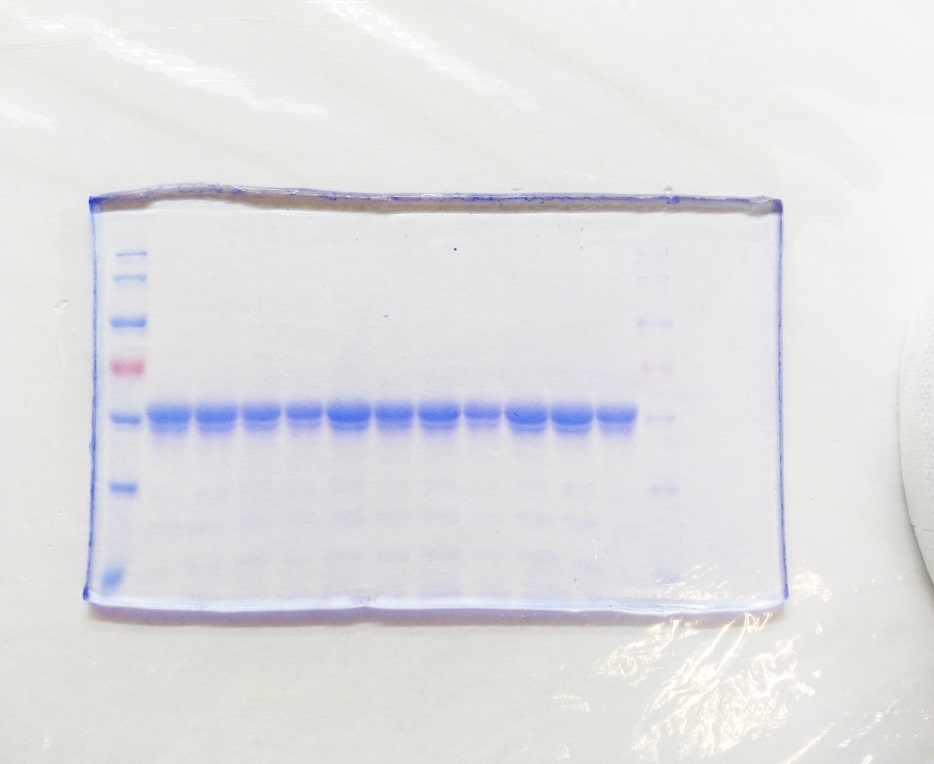
**

Coomassie blue staining, Comp1-9, Fig1B

**
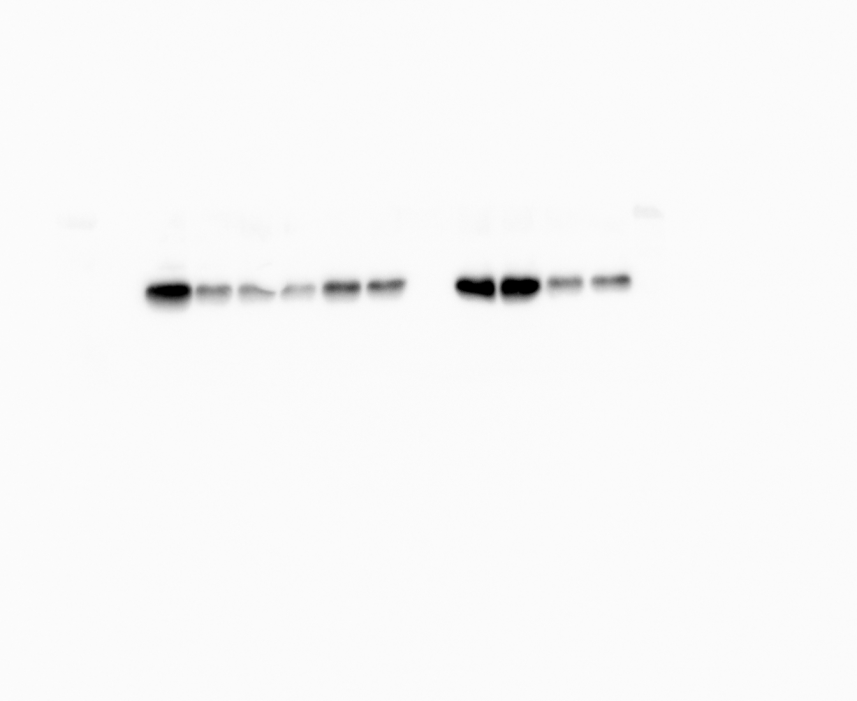

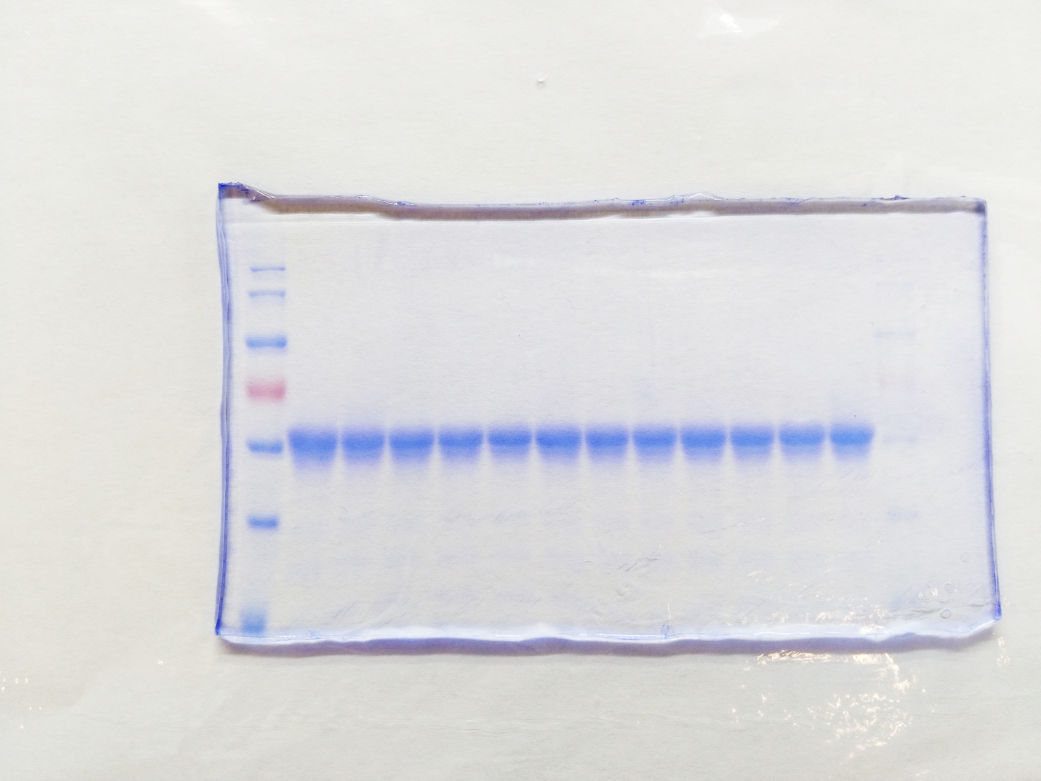
**

WB, Steptavidin-HRP-Comp10-19, Fig1B

Coomassie blue staining, Comp1-9, Fig1B

**Figure 2**

**
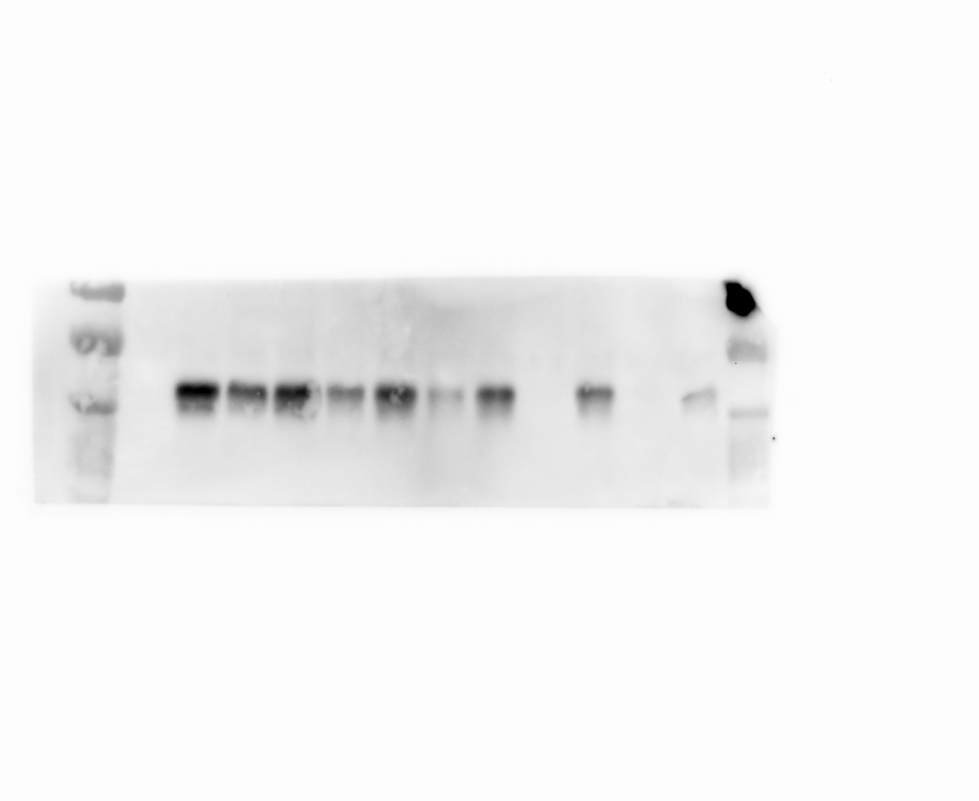

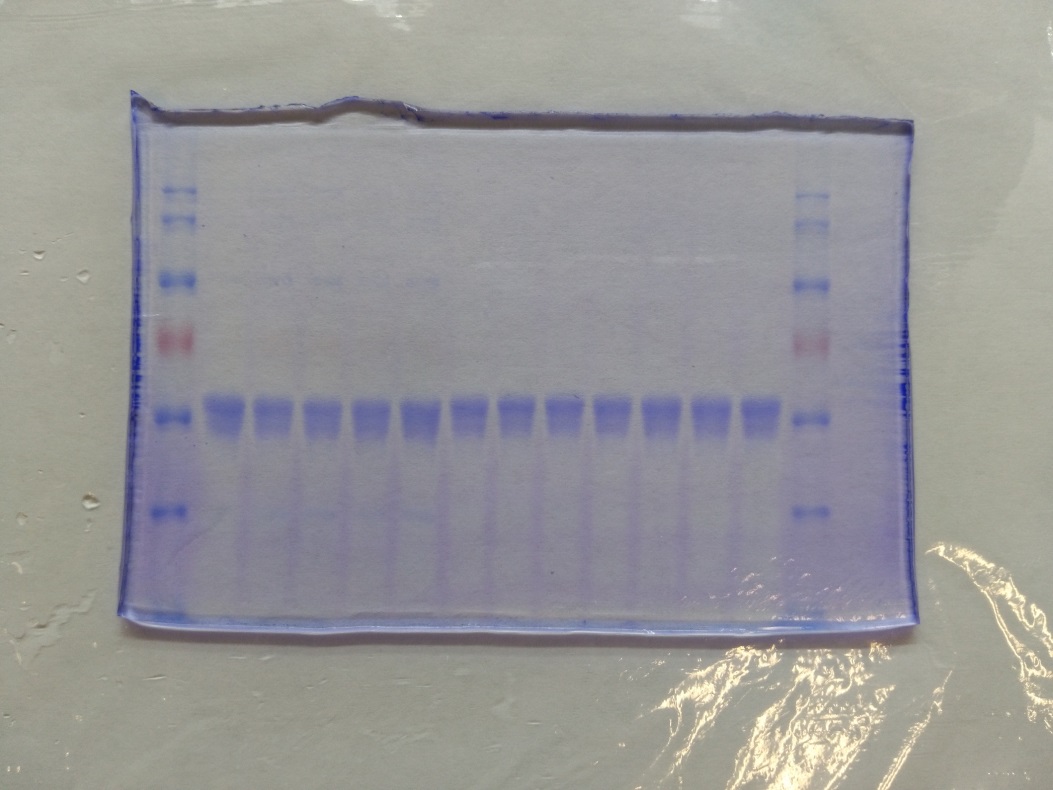
**

Coomassie blue staining, Fig2B

WB, Steptavidin-HRP, Fig2B

**Figure 3**

**
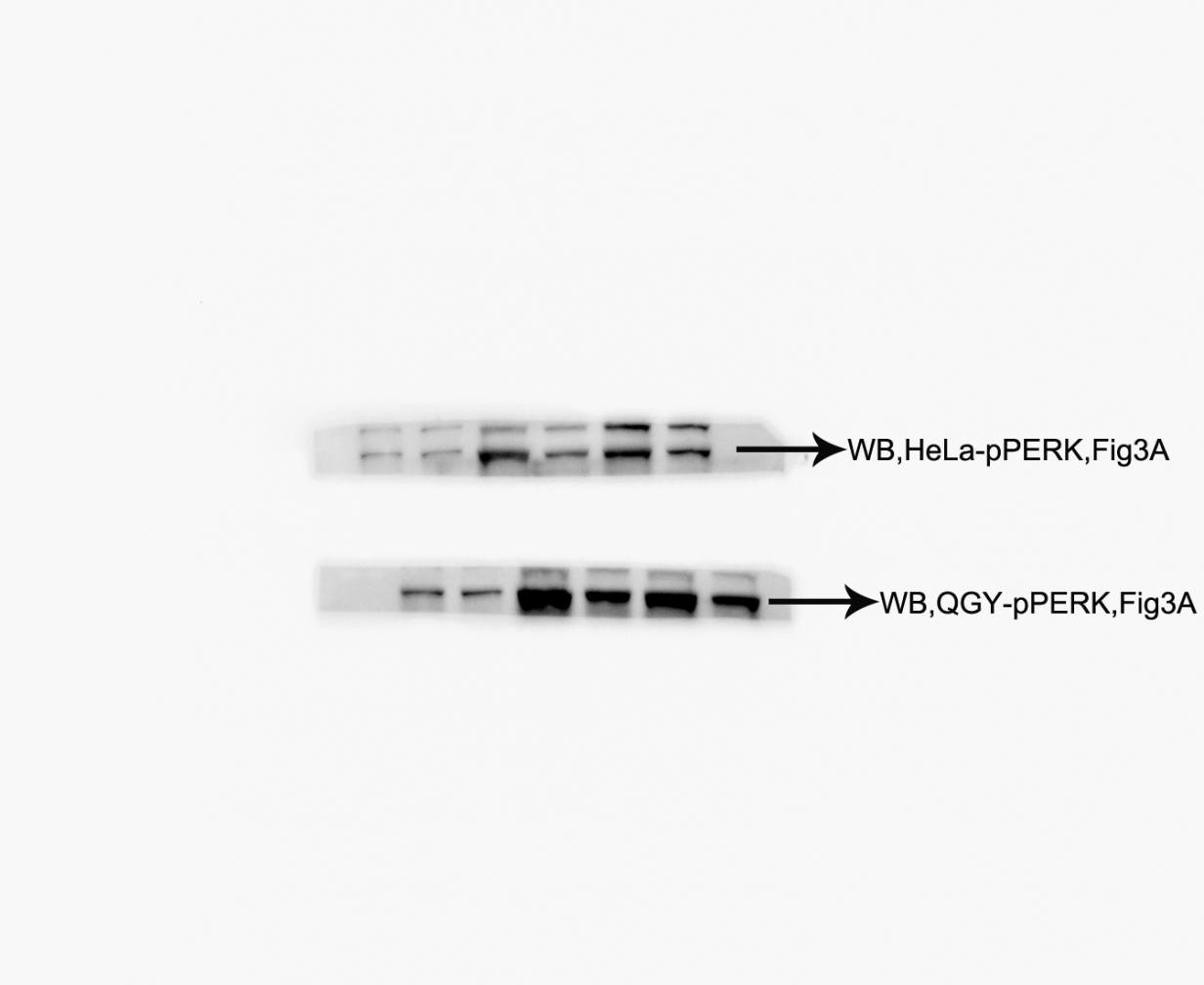

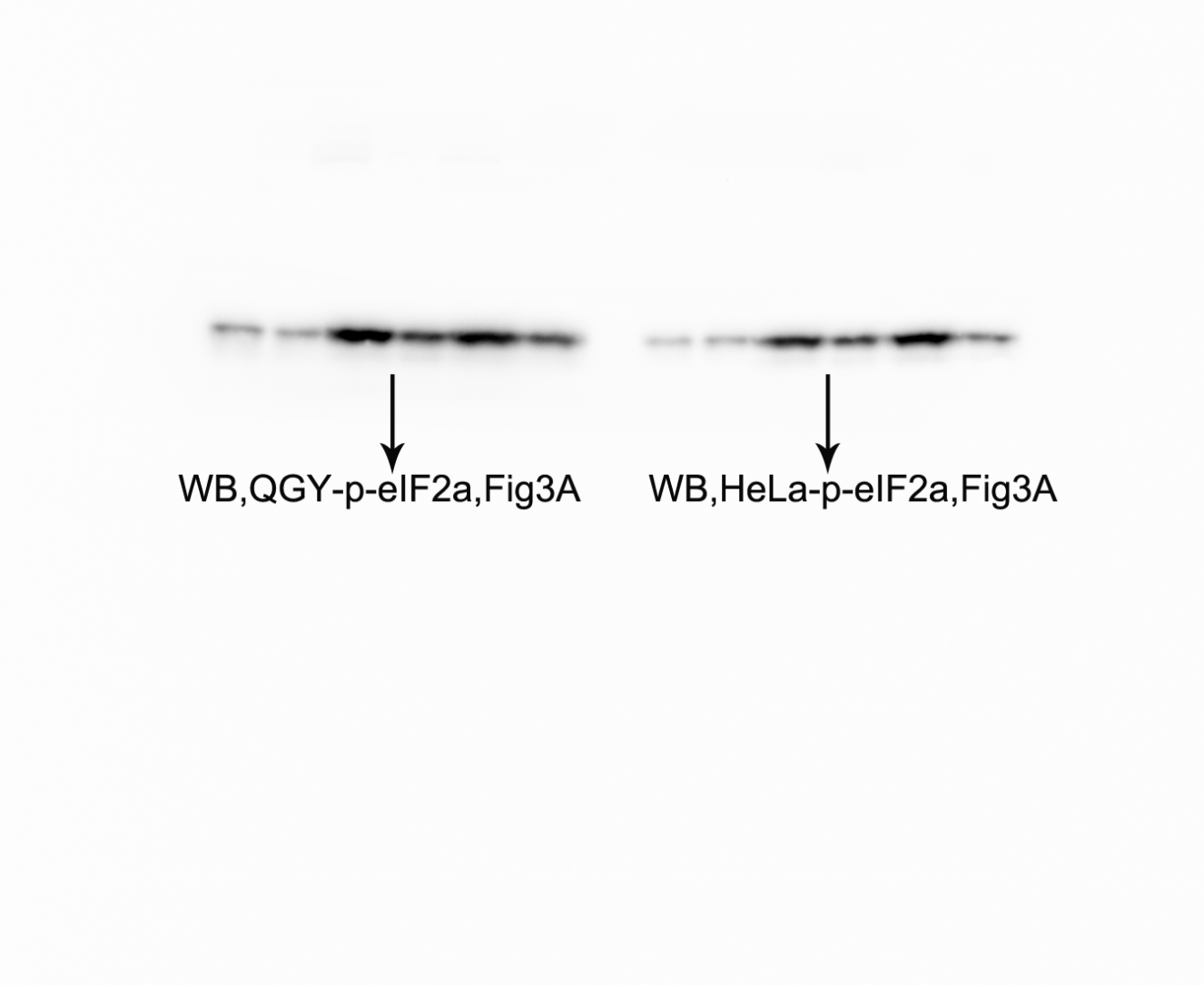

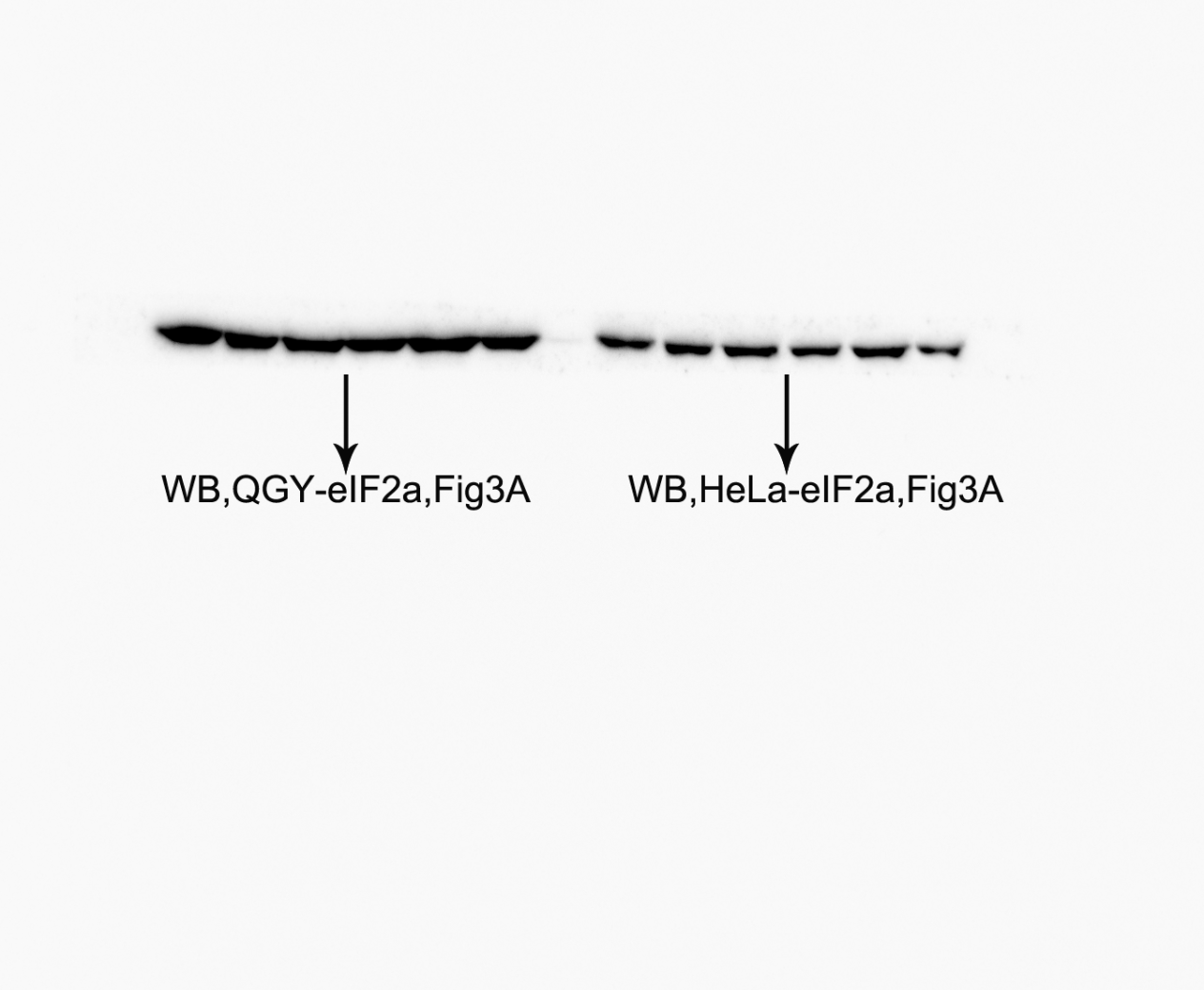

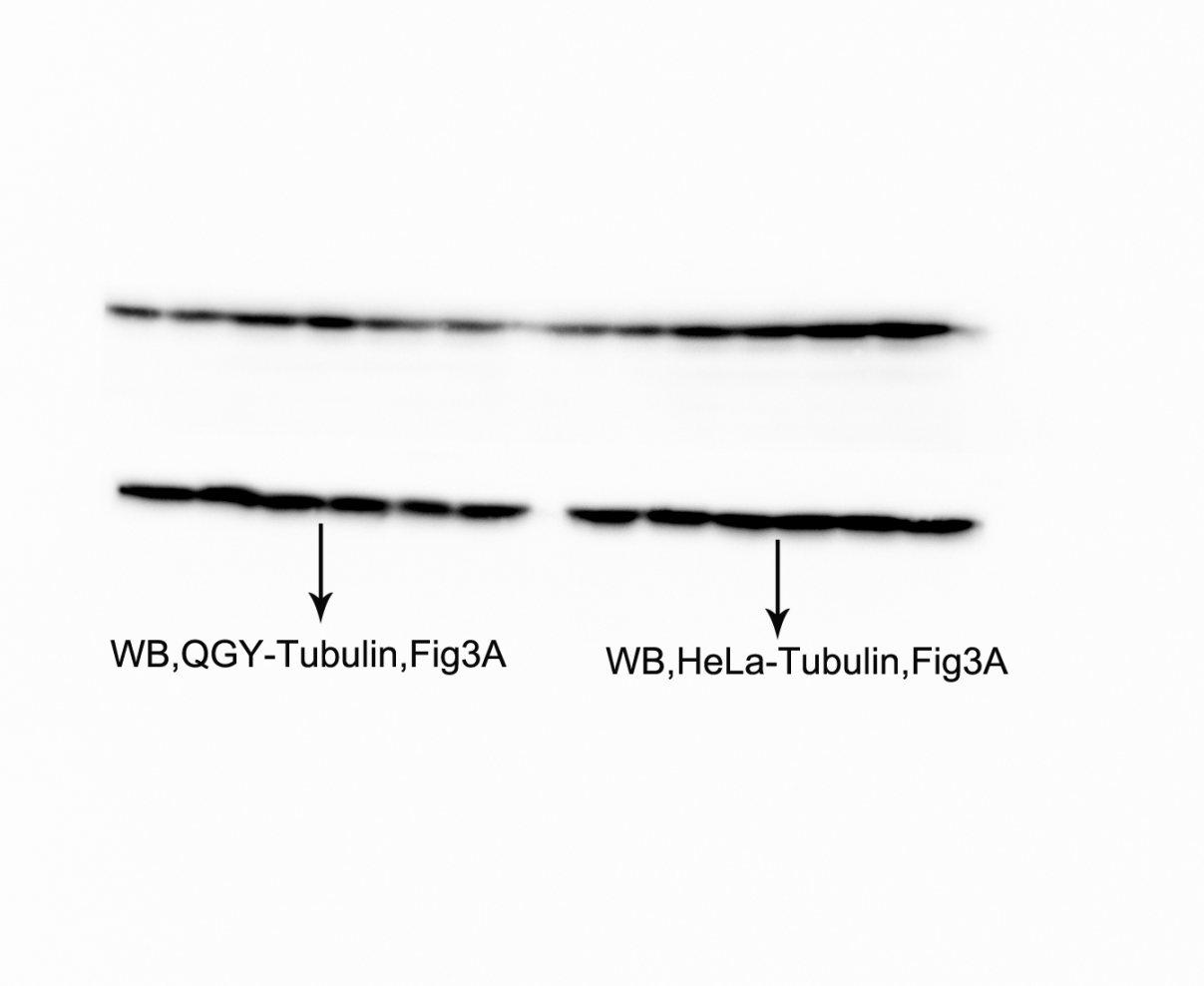
**

**Figure S1**

**
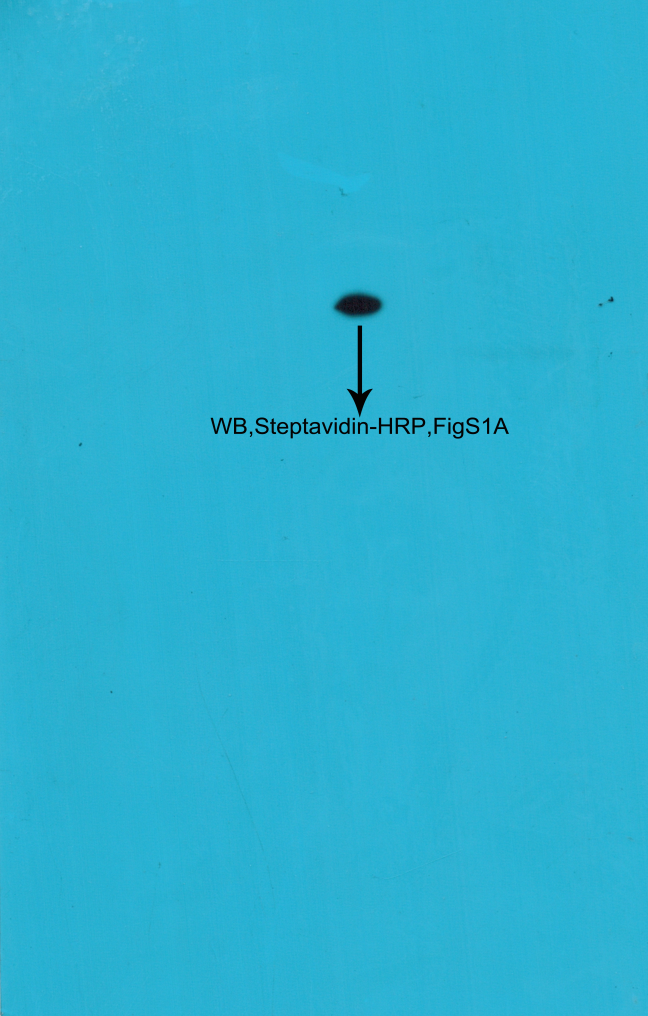

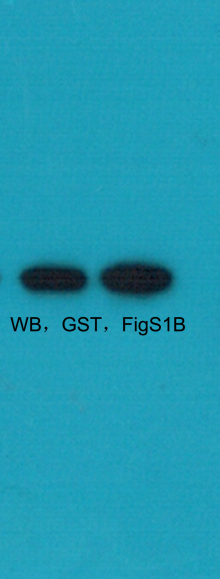
**

**Figure S2**

**
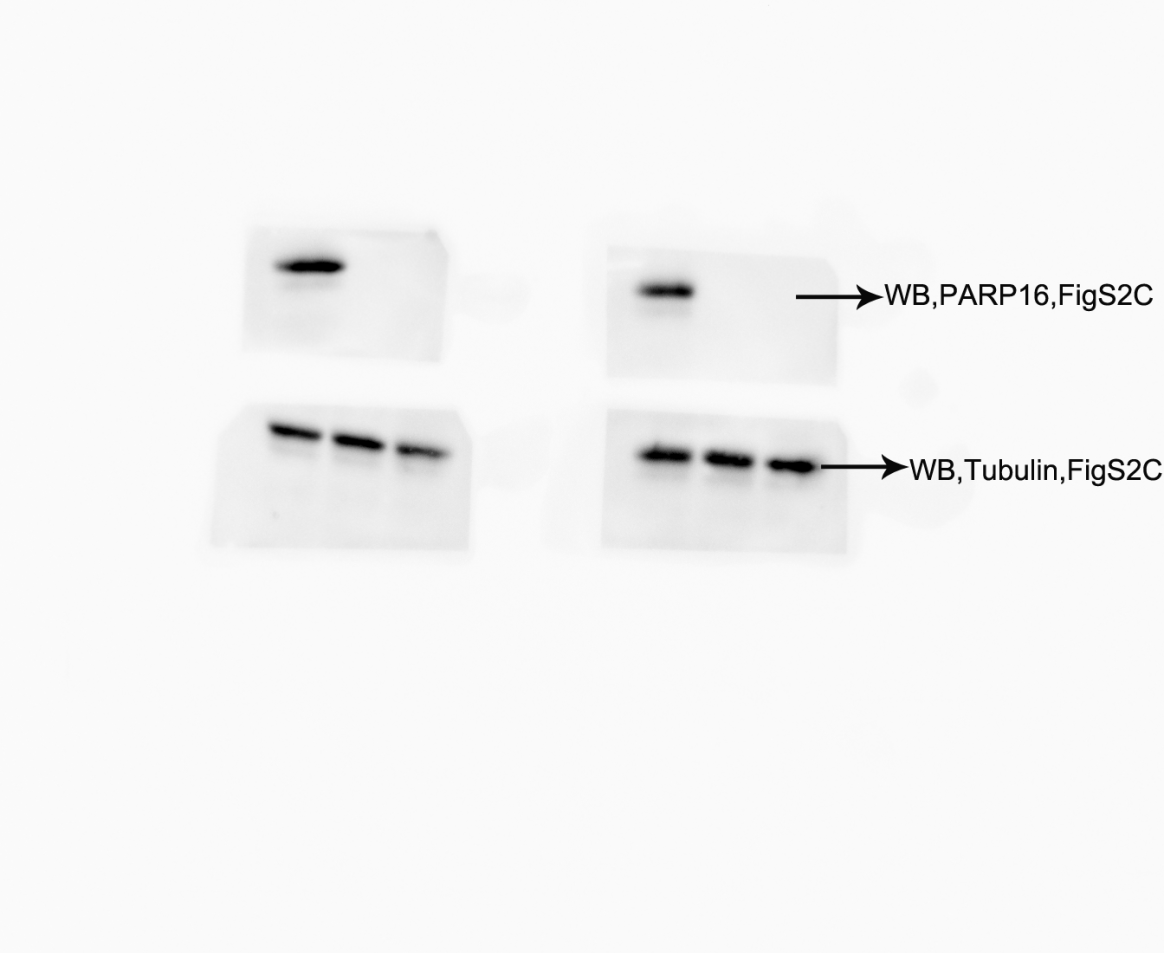

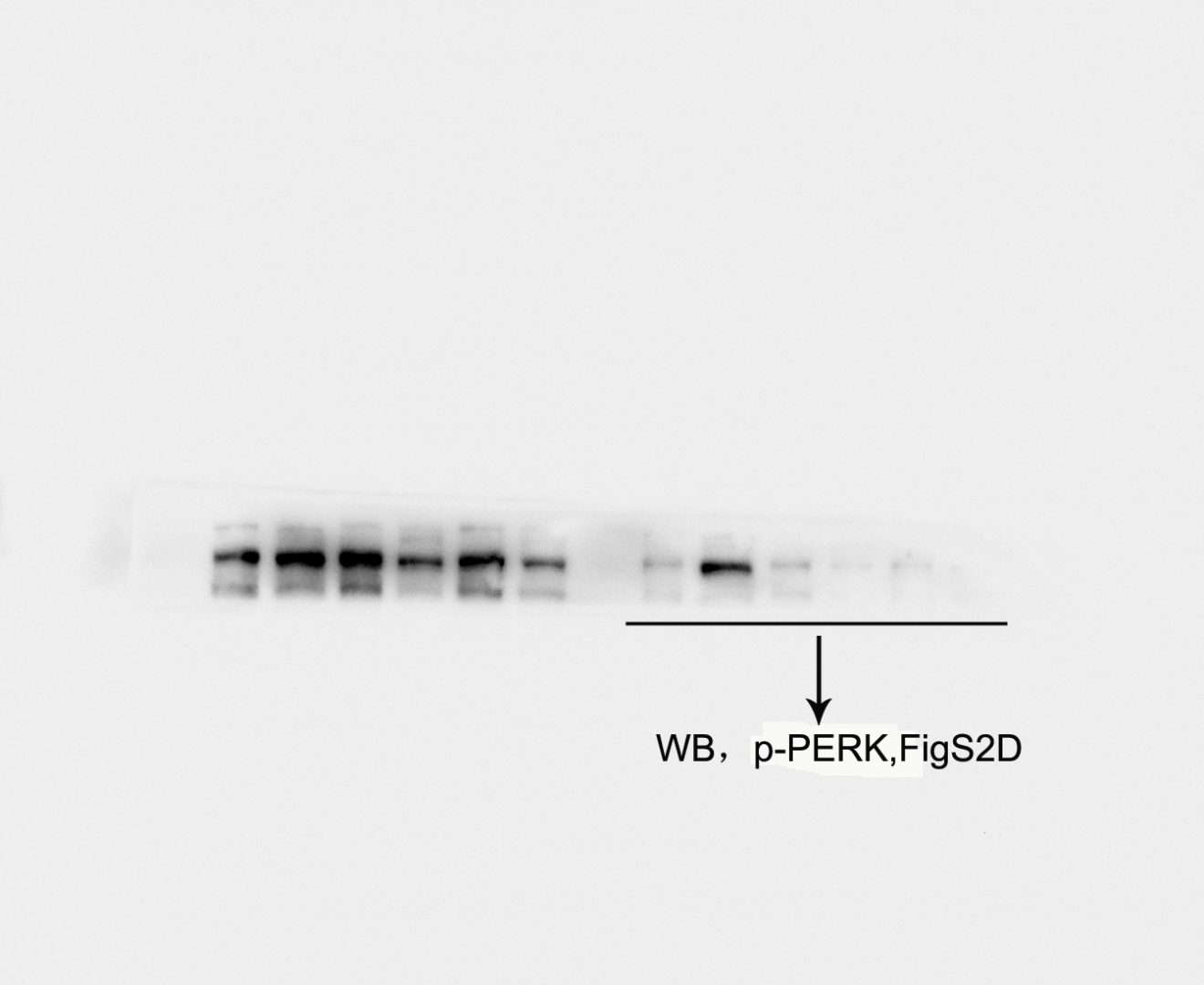

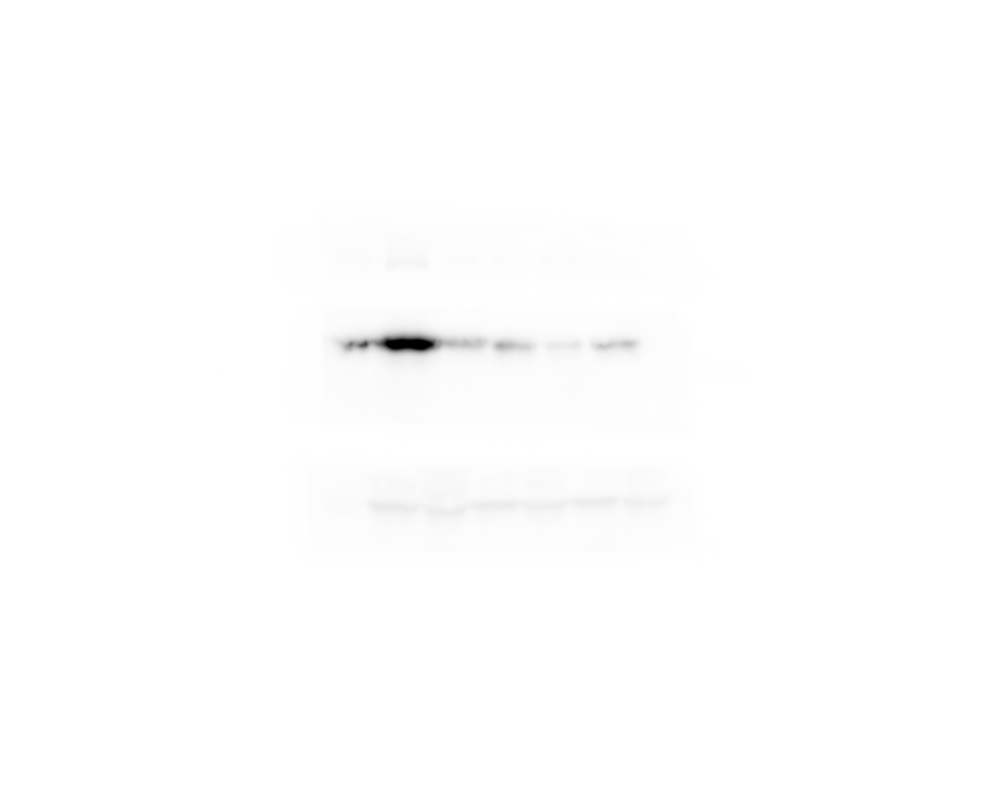

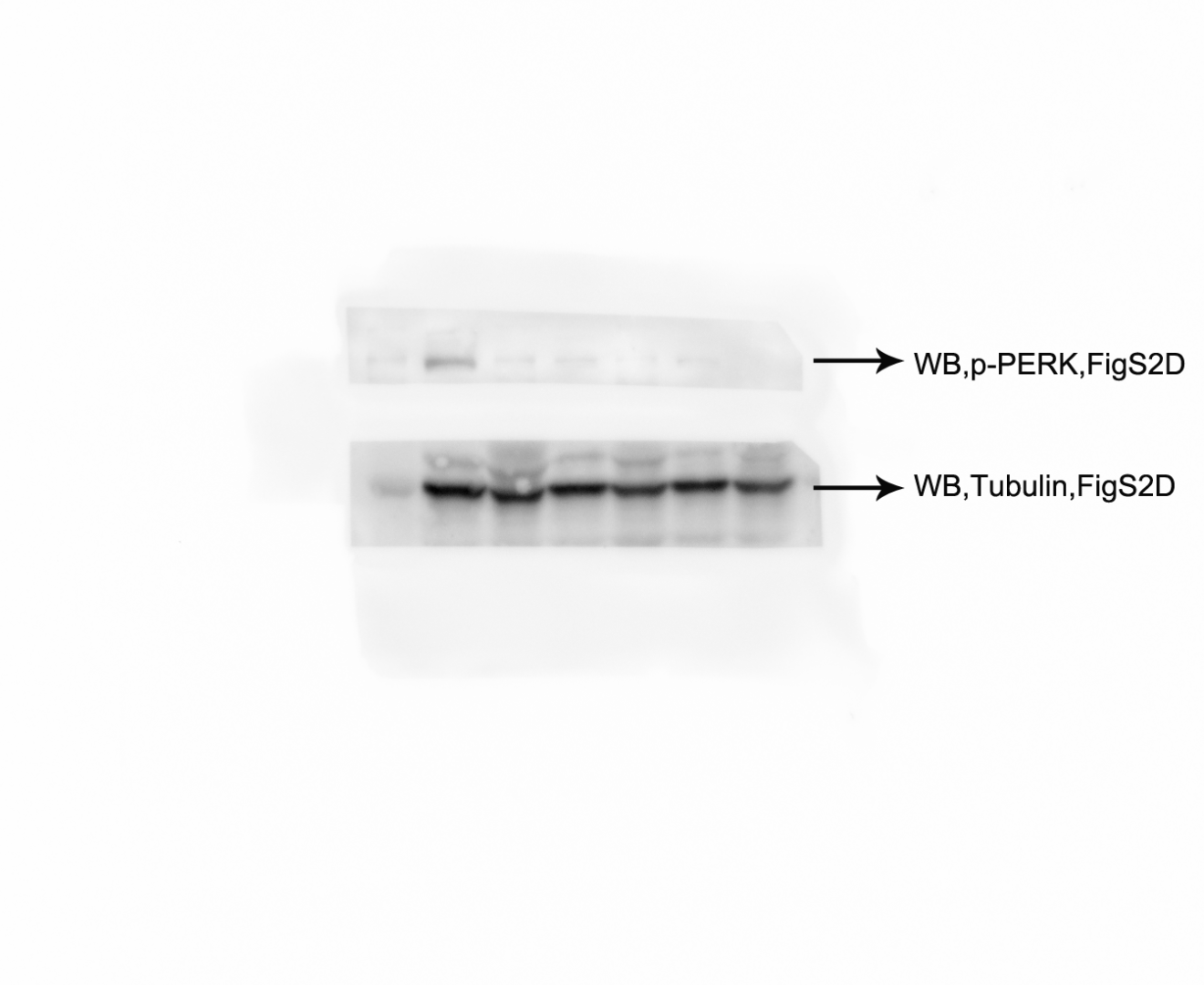
**

WB, p-eIF2a, FigS2D
